# Anesthetic Management in a Patient with Dandy-Walker Syndrome Undergoing Supraglottoplasty and Adenotonsillectomy: A Case Report

**DOI:** 10.7759/cureus.95994

**Published:** 2025-11-03

**Authors:** Ahmed Alanzi, Dawood Alatefi, Abdulrahman A Alselaiti, Keith Johnston, Shahid Adeel

**Affiliations:** 1 Anesthesia and Pain Management Departement, King Hamad University Hospital, Muharraq, BHR; 2 Neurological Surgery, Center for Spinal Surgery, Werner-Wicker Clinic, Bad Wildungen, DEU

**Keywords:** airway management, dandy–walker malformation, laryngomalacia, obstructive sleep apnea, pediatric anesthesia, supraglottoplasty

## Abstract

We report the case of a three-year-old girl with Dandy-Walker syndrome (DWS), severe obstructive sleep apnea (OSA), micrognathia, laryngomalacia, seizure disorder, and recurrent aspiration pneumonia who was scheduled for direct laryngotracheal bronchoscopy, supraglottoplasty, and adenoidotonsillectomy. Preoperative optimization included bronchodilator therapy and pulmonology input. Anesthetic management prioritized spontaneous ventilation using inhalational induction with sevoflurane followed by propofol infusion. Airway topicalization with lidocaine and adjunctive dexmedetomidine facilitated atraumatic airway manipulation. After endoscopic airway assessment, tracheal intubation was performed to complete the surgery. Given the extensive airway instrumentation and severe OSA, the patient remained intubated postoperatively and was extubated uneventfully in the pediatric intensive care unit after 24 hours. This case highlights the challenges of anesthetic management in children with complex syndromic airways and emphasizes the importance of strategies that maintain spontaneous ventilation, attenuate airway reflexes, and allow for safe postoperative monitoring.

## Introduction

Dandy-Walker syndrome (DWS) is a rare congenital malformation of the posterior fossa characterized by hypoplasia of the cerebellar vermis, cystic dilatation of the fourth ventricle, and enlargement of the posterior fossa [1]. This triad often results in macrocephaly, delayed neurodevelopment, and signs of increased intracranial pressure, such as vomiting, irritability, and ataxia [2]. Associated anomalies, including craniofacial dysmorphisms (e.g., micrognathia, cleft palate) and neuromuscular deficits, further complicate anesthetic management [3]. Cognitive impairment and seizure disorders are also common, reflecting the extent of underlying cerebral dysgenesis [4]. Airway anomalies such as micrognathia, glossoptosis, and hypotonia predispose these children to obstructive sleep apnea (OSA), which significantly increases the risk of perioperative respiratory complications [5,6]. Managing such patients requires an approach that balances neuroprotection, airway safety, and hemodynamic stability. Here, we describe the anesthetic management of a three-year-old girl with DWS, severe OSA, and seizure disorder undergoing direct laryngotracheal bronchoscopy, supraglottoplasty, and adenoidotonsillectomy. This case highlights the multidisciplinary challenges of securing a difficult pediatric airway while mitigating the risks of intracranial pressure elevation and postoperative respiratory compromise.

## Case presentation

**Figure 1:**
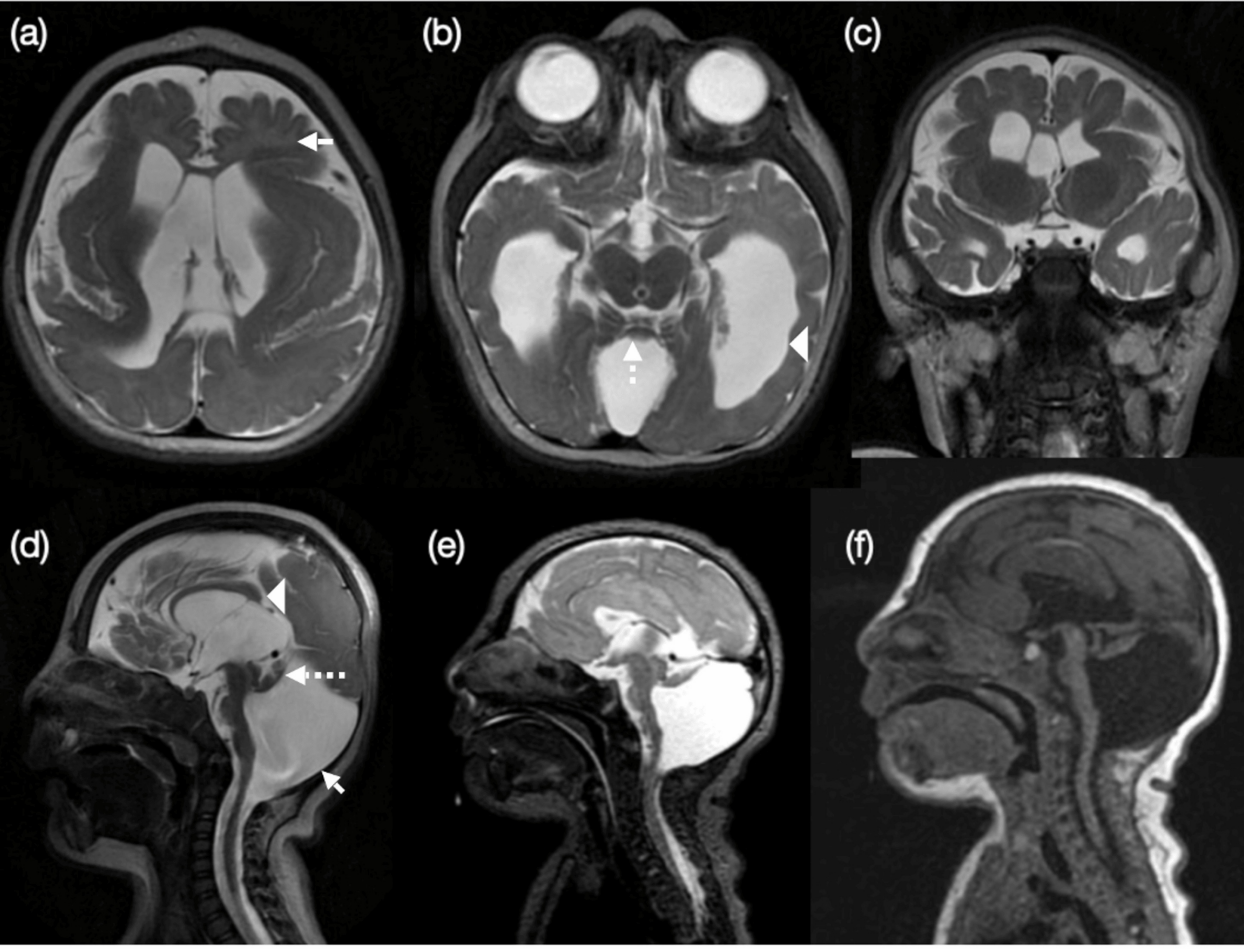
MRI brain (axial, coronal, sagittal) showing cystic dilatation of the fourth ventricle with enlarged posterior fossa (d, solid arrow) and hypoplastic, cephalad-rotated vermis (d, dashed arrow). Findings include severe microcephaly (the craniofacial index is severely low for age) with global white matter loss (a, solid arrow), simplified gyral pattern, colpocephalic lateral ventricles (b, arrow head) with preserved corpus callosum (d, arrow head), brainstem atrophy, and underopercularized Sylvian fissures.

A three-year-old female, a known case of DWS, was scheduled for direct laryngotracheal bronchoscopy with supraglottoplasty and adenoidotonsillectomy under general anesthesia. She was born full-term at 38 weeks via spontaneous vaginal delivery. Her medical history included hyperreactive airway disease, seizure disorder, intermittent stridor, and congenital glaucoma. The child experienced generalized tonic-clonic seizures approximately twice monthly during the first year of life, well controlled with levetiracetam in the six months preceding surgery. The diagnosis of DWS was associated with severe microcephaly, global developmental delay, and feeding difficulties (Figure 1). She had multiple previous hospital admissions due to status epilepticus and aspiration pneumonia. There was no history of drug allergies. Her regular medications included levetiracetam (Keppra) and clonazepam (Rivotril). The patient had undergone previous orthopedic surgery and trabeculotomy for congenital glaucoma, both of which were uneventful.

**Figure 2:**
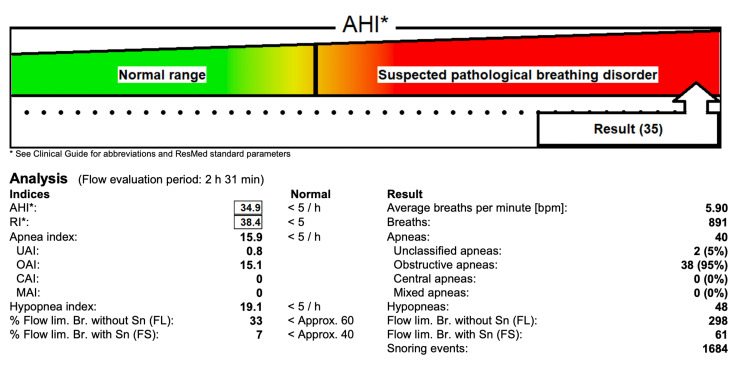
Preoperative sleep study demonstrating severe obstructive sleep apnea. this is a picture directly taken from the lab report done in our hospital.

Preoperative assessment revealed a weight of 12 kg and a height of 92 cm. She had micrognathia but adequate mouth opening. Respiratory examination demonstrated inspiratory stridor and wheeze, which responded to bronchodilator therapy. Cardiac examination was unremarkable. Laboratory investigations were within normal limits, including hemoglobin (12.1 g/dL; normal 11.5-15.5), white blood cell count (7.8 × 10⁹/L; normal 4.0-11.0), platelets (280 × 10⁹/L; normal 150-400), sodium (138 mmol/L; normal 135-145), potassium (4.1 mmol/L; normal 3.5-5.1), and creatinine (0.5 mg/dL; normal 0.3-0.7). The apnea-hypopnea index (AHI), a standard polysomnographic metric defined as the number of apneas and hypopneas per hour of sleep, was used to quantify the severity of OSA and was 35 [7] (Figure 2).

Her respiratory condition was optimized in consultation with a pediatric pulmonologist, and she received nebulization therapy one hour prior to surgery. In the operating room, standard ASA monitors were applied. A 24-G intravenous cannula was already in place from the ward. The operating room temperature was maintained at 24°C.

All necessary equipment was prepared, including a functional anesthesia machine, a difficult airway cart, and emergency medications such as epinephrine (1 mcg/mL), atropine (0.1 mg/mL), and succinylcholine (10 mg/mL). The anesthesia plan prioritized the maintenance of spontaneous respiration throughout the procedure. Anesthesia was induced with sevoflurane 8% in 100% oxygen, combined with a propofol infusion at 200 mcg/kg/min. Dexamethasone 0.5 mg/kg was administered to reduce airway edema, and glycopyrrolate 10 mcg/kg was given to manage secretions. A jaw thrust maneuver was performed to assess the depth of anesthesia. Once adequate depth was confirmed, 2 mL of 2% lidocaine was sprayed over the arytenoids, vocal cords, and trachea.

**Figure 3:**
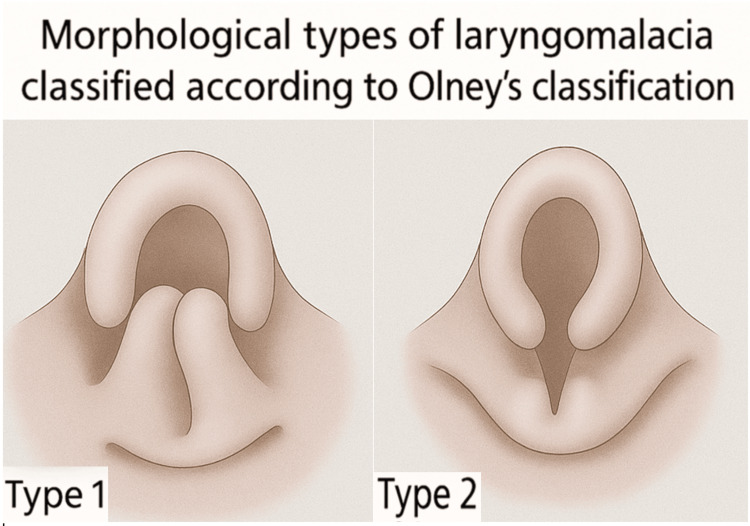
Illustrative schematic showing the two subtypes of laryngomalacia according to the classification by Olney et al. Type 1 demonstrates prolapsing supra-arytenoid tissue narrowing the glottic inlet, whereas type 2 shows shortened aryepiglottic folds and a long, omega-shaped epiglottis that curls on itself. Created de novo by the authors using Adobe Firefly (Adobe Inc., San Jose, CA, USA) [9], based on the classification described by Olney et al. [8].

The patient was then handed over to the pediatric ENT team. Prior to scope insertion, intravenous dexmedetomidine 4 mcg was administered. Airway assessment revealed redundant arytenoid mucosa (type 1) and shortened aryepiglottic folds (type 2) (Figure 3) [8]. The vocal cords were mobile bilaterally with spontaneous breathing, and there was no evidence of tracheomalacia or subglottic lesions. Endotracheal intubation was performed using a size 4.5 cuffed tube to proceed with the supraglottoplasty and adenoidotonsillectomy. The surgical procedure lasted approximately one hour, during which the patient received 120 mL of Ringer's lactate. Intraoperatively, standard ASA monitors were supplemented with continuous end-tidal CO₂ (EtCO₂) and bispectral index (BIS) monitoring. EtCO₂ was maintained between 35 and 40 mmHg to ensure normocapnia and avoid intracranial pressure surges, while BIS was maintained between 45 and 60 to provide adequate anesthesia depth and reduce the risk of intraoperative seizures. Due to significant airway manipulation, a decision was made to keep the patient intubated postoperatively for 24 hours. She was successfully extubated the following day in the pediatric intensive care unit without complications.

## Discussion

Neuroanesthetic considerations

DWS involves central nervous system malformations that profoundly affect anesthesia care. The hallmark imaging findings are cerebellar vermian hypoplasia with upward rotation and enlargement of the fourth ventricle [10]. Hydrocephalus and macrocephaly often coexist, predisposing patients to elevated ICP. Many children with DWS have developmental delay, poor coordination, and seizure disorders [11]. In our patient, a history of seizures and a bulging fontanel indicated prior ICP elevations. Thus, controlling ICP intraoperatively was critical. In this case, propofol was preferred for induction and maintenance since it reduces cerebral blood flow and ICP [12].

Airway challenges

Craniofacial anomalies are common in DWS, often including micrognathia and a high-arched palate. Micrognathia reduces mandibular space, which makes laryngoscopy and mask ventilation difficult [12]. In the literature, DWS patients have required fiberoptic or alternative techniques due to anterior laryngeal position and jaw hypoplasia [13]. Redundant pharyngeal tissue from OSA and potential laryngomalacia can cause stridor; thus, a surgeon needs to be immediately available for emergency airway intervention or tracheostomy. Our patient’s polysomnogram showed an AHI of 35, classifying as severe pediatric OSA. Severe OSA multiplies perioperative risk. For example, Thampi et al. suggested that children with severe OSA have significantly higher rates of desaturation, laryngospasm, and unplanned ICU admission, with 35% of severe OSA children experiencing perioperative respiratory events compared to 9% with mild OSA [14]. Moreover, OSA patients are more sensitive to sedatives and opioids; residual anesthetics can precipitate upper airway collapse [15]. Therefore, it is important to maintain vigilant monitoring and minimize respiratory depressants during anesthesia in such patients.

Pharmacological strategy

**Figure 4:**
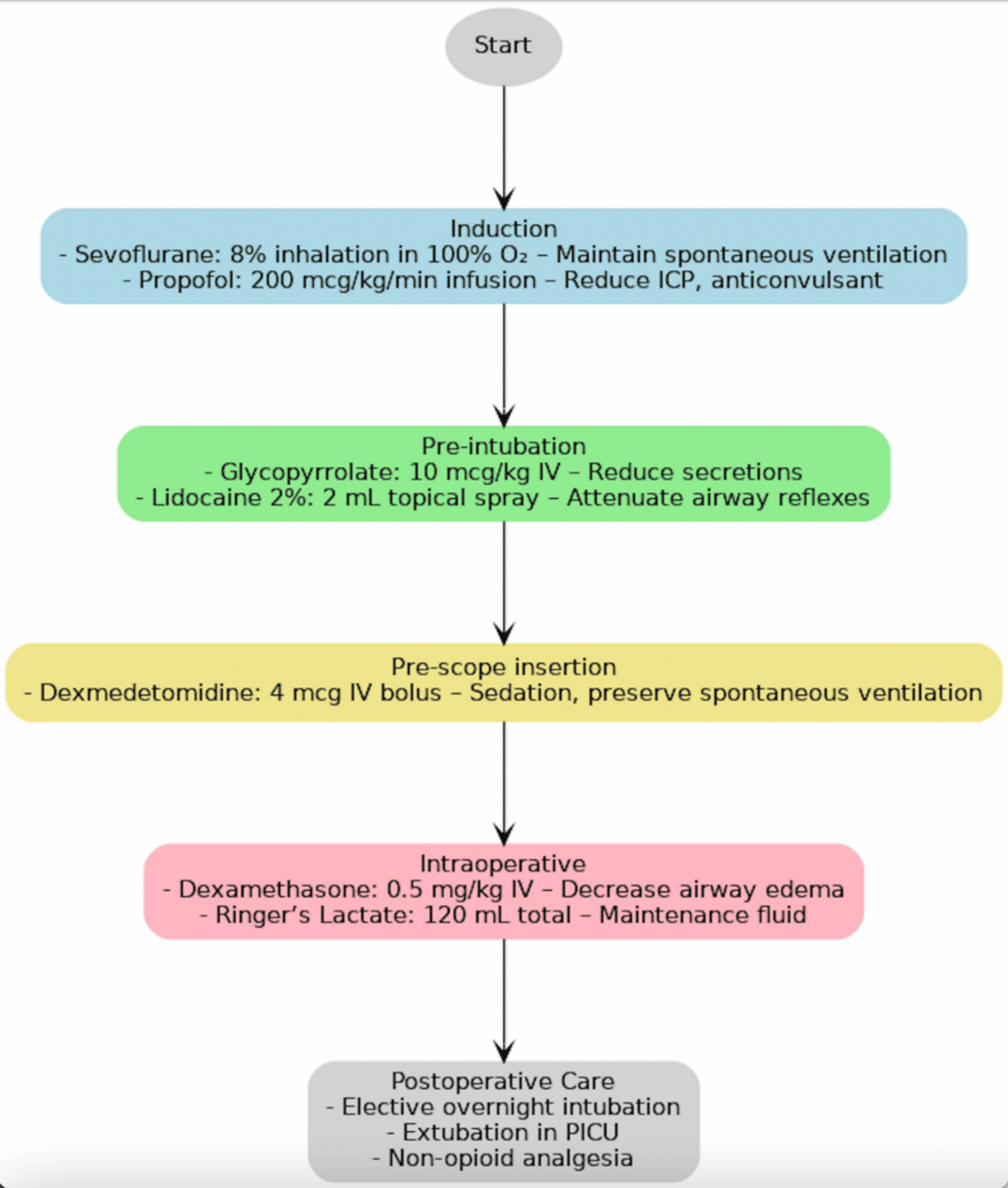
Timeline of key stages (induction → pre-intubation → pre-scope → intraoperative → postoperative), listing the medication at each step with administered dose and intent. Abbreviations: ICP, intracranial pressure; PICU, pediatric intensive care unit; IV, intravenous; O₂, oxygen. Doses are weight-based; adapt to patient weight and local protocols

Given the predicted airway difficulties and OSA, we favored an inhalation induction with sevoflurane in oxygen to maintain spontaneous ventilation while securing IV access. This is consistent with the case report by Quezado et al., who also used a similar protocol for complex microlaryngeal surgery [16]. After IV placement, we transitioned to a propofol infusion to control ICP and seizures and initiated a dexmedetomidine infusion for sedation. Dexmedetomidine is particularly useful in pediatric airway surgery, as it provides sedation while preserving respiratory drive and blunting airway reflexes [17]. As demonstrated in complex microlaryngeal surgeries, combining dexmedetomidine with propofol can obviate the need for endotracheal intubation entirely [16]. In our case, we did intubate but used dexmedetomidine to facilitate tolerance of the airway and minimize emergence agitation. To minimize laryngeal stimulation and coughing, a topical lidocaine spray was applied to the glottis before instrumentation. Evidence supports both IV and topical lidocaine for laryngospasm prevention in children [18]. The complete perioperative medication regimen, including doses and timing, is summarized in Figure 4.

Airway device selection

The use of a cuffed 4.5-mm endotracheal tube was deliberate. Modern pediatric cuffed tubes provide a reliable seal and reduce the need for tube exchanges without increasing post-extubation stridor, as evidenced by a meta-analysis of six studies [19].

Seizure management

The patient’s history of epilepsy necessitated careful planning. All anticonvulsants were continued throughout the perioperative period. Propofol, besides lowering ICP, has intrinsic anticonvulsant properties, which were advantageous during induction [20].

Aspiration risk

Children with DWS and severe OSA often have feeding difficulties and gastroesophageal reflux, increasing aspiration risk. Although no large-scale studies have quantified aspiration pneumonia incidence specifically in DWS, there is growing evidence of high rates of dysphagia in patients with posterior fossa and brainstem pathology. A recent meta-analysis of posterior fossa tumor resections reported a pooled dysphagia prevalence of 21.7 % (95% CI 16.9-26.6) across 22 studies of over 20,900 patients [21]. In pediatric cohorts, Wright et al. (Alder Hey experience) also identified a notable incidence of dysphagia following posterior fossa surgery in children [22]. Given the anatomical and functional overlap between posterior fossa lesions and the neuroanatomy involved in Dandy-Walker, these findings reinforce the plausibility of aspiration risk in Dandy-Walker patients, especially when compounded by hypotonia, brainstem involvement, or reflux. Nonetheless, future prospective studies focused on DWS are needed to define the true incidence of aspiration pneumonia in this population

Postoperative considerations

Given the severe OSA and neurodevelopmental delays, we avoided routine premedication or sedation. Sedatives such as midazolam or chloral hydrate can exacerbate airway obstruction and cause paradoxical agitation in neurologically impaired children. In our patient, hyperactive airway reflexes and baseline tone irregularity could have been worsened by sedatives. Instead, we used non-pharmacological calming strategies (parental presence, music) in the induction area. We elected a cuffed 4.5-mm endotracheal tube for ventilation. Modern pediatric guidelines support cuffed tubes in children of this age, as they can reduce gas leak without increasing airway injury. The meta-analysis by Zhang et al. found that cuffed tubes significantly decreased the need for tube exchanges in children, with no increase in post-extubation stridor or complications [19]. Dexmedetomidine proved invaluable for airway management in this case. This α₂-agonist provides sedation and analgesia without significant respiratory depression. Mahmoud et al. noted that dexmedetomidine uniquely obtunds airway reflexes while preserving spontaneous ventilation and hemodynamic stability [17]. Micrognathia necessitated specific ventilation techniques. Preoxygenation was performed with a two-person mask ventilation approach. Postoperative analgesia was approached cautiously. Non-opioid analgesics were emphasized: regular weight-based acetaminophen and low-dose NSAIDs (ibuprofen), unless contraindicated. We avoided regional nerve blocks, as the patient’s developmental delay made cooperation and consistent block evaluation difficult; the surgical site (airway) was also not amenable to blocks.

Comparison with the literature

Our experience aligns with published reports of DWS in anesthesia. For example, Jang et al. described an adolescent DWS patient with macrocephaly and micrognathia, making intubation “challenging” [2]. Like them, we noted difficult airway anatomy and emphasized ICP control with propofol. Other case reports similarly emphasize the need to evaluate airway anatomy and plan for difficult intubation in DWS [2,23]. Overall, the literature supports our multimodal approach: anticipate challenges, choose neuroprotective anesthetics, and prepare for postoperative ICU care [2,24].

## Conclusions

This case underscores the importance of meticulous perioperative planning and individualized anesthetic management in children with DWS, particularly when coexisting airway abnormalities and OSA increase anesthetic risk. A multidisciplinary approach integrating anesthesia, otolaryngology, and neurology was essential to achieving optimal intraoperative stability and safe postoperative recovery. The patient had an uneventful recovery with no postoperative respiratory or hemodynamic complications, highlighting the value of individualized anesthetic planning and coordinated teamwork in DWS.

## References

[1] Ocampo-Navia MI, Perez-Mendez W, Rodriguez-Alvarez MP, Chadid-Contreras J, Vergara MF (2025). Dandy-Walker syndrome: an updated literature review. Childs Nerv Syst.

[2] Jang JS, Lee JJ, Park WJ, Kim EY, Lim SY (2013). Anesthetic management of an adolescent with Dandy-Walker syndrome. Korean J Anesthesiol.

[3] Oria MS, Rasib AR, Pirzad AF, Wali Ibrahim Khel F, Ibrahim Khel MI, Wardak FR (2022). A rare case of Dandy-Walker syndrome. Int Med Case Rep J.

[4] Almadani A, Mohammed A, Algalil AA (2023). A Sudanese pediatric patient with Dandy-Walker syndrome, epilepsy, and hemophilia: a case report. J Neurol Sci.

[5] Mehta B, Waters K, Fitzgerald D, Badawi N (2024). Clinical characteristics, associated comorbidities and hospital outcomes of neonates with sleep disordered breathing: a retrospective cohort study. BMJ Paediatr Open.

[6] Riaz A, Malik HS, Fazal N, Saeed M, Naeem S (2009). Anaesthetic risks in children with obstructive sleep apnea syndrome undergoing adenotonsillectomy. J Coll Physicians Surg Pak.

[7] Berry RB, Brooks R, Gamaldo C (2017). AASM scoring manual updates for 2017 (version 2.4). J Clin Sleep Med.

[8] Olney DR, Greinwald JH Jr, Smith RJ, Bauman NM (1999). Laryngomalacia and its treatment. Laryngoscope.

[9] (2025). Adobe Firefly. Adobe Inc., San Jose, CA, USA. https://firefly.adobe.com.

[10] Zamora EA, Das JM, Ahmad T (2023). Dandy-Walker malformation. StatPearls [Internet].

[11] Hayat F, Ismail M, Alqhtani MM (2023). Dandy-Walker syndrome: delayed acute presentation with unusual symptoms. Cureus.

[12] Johnston AJ, Steiner LA, Chatfield DA (2003). Effects of propofol on cerebral oxygenation and metabolism after head injury. Br J Anaesth.

[13] Buget MI, Edipoglu IS, Cemaller E, Eren S, Kucukay S (2015). Anesthetic management of a patient with Dandy-Walker syndrome for orthopedic surgery. J Med Cases.

[14] Thampi S, Chong SY, Pawar DK (2022). Perioperative adverse respiratory events in children with obstructive sleep apnoea. Airway.

[15] von Breunig F, Wappler F, Hagel C (2004). Histomorphologic examination of skeletal muscle preparations does not differentiate between malignant hyperthermia-susceptible and -normal patients. Anesthesiology.

[16] Burnett T, Bhalla T, Sawardekar A, Tobias JD (2011). Performance of the On-Q pain infusion device during changes in environmental temperature. Paediatr Anaesth.

[17] Mahmoud M, Mason KP (2015). Dexmedetomidine: review, update, and future considerations of paediatric perioperative and periprocedural applications and limitations. Br J Anaesth.

[18] Vagnoli L, Caprilli S, Messeri A (2010). Parental presence, clowns or sedative premedication to treat preoperative anxiety in children: what could be the most promising option?. Paediatr Anaesth.

[19] Brown TC (2013). The child in hospital--changes in the last 50 years. Paediatr Anaesth.

[20] Chiumello D, Pozzi T, Storti E, Caccioppola A, Pontiroli AE, Coppola S (2020). Body mass index and acute respiratory distress severity in patients with and without SARS-CoV-2 infection. Br J Anaesth.

[21] Duan Y, Wang Y, Zhang X, Huang J, Zhou Z, Zhao Q (2024). Prevalence of dysphagia following posterior fossa tumor resection: a systematic review and meta‑analysis. BMC Cancer.

[22] Morgan AT, Sell D, Ryan M, Raynsford E, Hayward R (2008). Pre and post-surgical dysphagia outcome associated with posterior fossa tumour in children. J Neurooncol.

[23] Burgess F, Fragoza K (2017). Fishing for answers in an ocean of data: The potential for big data analytics to enhance our knowledge of the complex regional pain syndromes. J Clin Anesth.

[24] Khezri MB, Oladi MR, Atlasbaf A (2012). Anesthetic considerations in pediatric patients with Dandy-Walker malformation: case series and literature review. Pediatr Neurosurg.

